# Surgical management of cystic echinococcosis—A 20-year case series and outcome analysis

**DOI:** 10.1371/journal.pntd.0013661

**Published:** 2026-04-24

**Authors:** Isabel Barreto, Ansgar Deibel, Jan Fehr, Beat Müllhaupt, Andreas E Kremer, Henrik Petrowsky, Jose Oberholzer, Jan Philipp Jonas

**Affiliations:** 1 Department of Visceral and Transplant Surgery, University Hospital Zurich, Zurich, Switzerland; 2 Department of Gastroenterology and Hepatology, University Hospital Zurich, University of Zurich, Zurich, Switzerland; Research Center for Hydatid Disease in Iran Kerman University of Medical Sciences, IRAN, ISLAMIC REPUBLIC OF

## Abstract

**Background:**

Cystic echinococcosis (CE) is a parasitic zoonosis, caused by the larval stage of *Echinococcus granulosus*, typically forming cysts in the liver. If untreated, CE can be life-threatening. Albendazole remains the standard medical therapy, but surgical intervention is indicated in selected cases.

**Methodology/principal findings:**

We conducted a retrospective, multicenter outcome analysis in Switzerland of surgically treated cystic echinococcosis from 2004 to 2024. Patients with CE stages 1-3b undergoing pericystectomy, endocystectomy, or non-resectional procedures were included. The primary objective was to evaluate the safety and efficacy of surgical treatment. Data on patient demographics, disease characteristics, and perioperative morbidity and mortality, as well as recurrence rates stratified by surgical technique were analyzed.

Among 37 patients, 83.8% (n = 31) had hepatic involvement, 8.1% (n = 3) pulmonary, 5.4% (n = 2) intestinal, and 2.7% (n = 1) psoas involvement. All patients received adjuvant albendazole therapy for a median duration of 4 months (3–204 months). Across 39 surgical interventions, 82.1% of procedures (n = 32) had one or two cysts (range 1–8), with a median cyst size of 8 cm (2.1–19 cm). The median follow-up was 93 months (12–230 months). The overall recurrence rate at the procedure level was 7.7% (n = 3), occurring after three different procedures (endocystectomy, cyst aspiration and fenestration, and open PAIR with cyst deroofing). No recurrences were observed after pericystectomy (0/32 procedures). The 90-day postoperative mortality rate was 0%.

**Conclusions:**

In this cohort, surgical treatment, particularly pericystectomy, was associated with low recurrence and no 90-day mortality. These findings support surgery as an important component of multimodal management in appropriately selected patients.

## Author summary

Cystic echinococcosis is a parasitic disease that causes cystic lesions, most often in the liver. Although antiparasitic medication is important, surgery remains the only curative option in many cases. A key surgical question involves the extent of resection: whether to remove only the cyst contents and inner layers or to perform a complete excision of the cyst, including its pericystic wall. This decision has significant implications for radicality, preservation of healthy tissue, and the risk of recurrence. We conducted a multicenter retrospective analysis covering 20 years of surgical treatment at a Swiss tertiary referral center and partner hospitals. Among 37 patients undergoing 39 procedures, pericystectomy was the most common technique. This method achieved complete removal of the cyst and was associated with no recurrences during long-term follow-up, while more conservative approaches experienced isolated relapses. Overall, morbidity was low, and there was no procedure-related mortality. These findings support pericystectomy, combined with perioperative anthelmintic therapy, as an effective and safe standard for selected patients requiring surgery.

## Introduction

Cystic echinococcosis (CE) is a zoonosis caused by the larval tissue of the dog tapeworm *Echinococcus granulosus* sensu lato, which consists of 10 genotypes and 5 species. These organisms are found globally, affecting both endemic and non-endemic regions through migration and population mobility [1,2]. In Switzerland, cystic echinococcosis is a notifiable disease, and confirmed cases are reported to the relevant public health authorities. Humans who become infected through fecal-oral transmission are considered accidental hosts [2–4]. The liver is the main organ affected, though cysts can develop in almost any organ, including the lungs, spleen, kidneys, and brain [3]. However, up to 80% of patients have involvement of a single organ, with a solitary cyst localized in the liver (80%) or lungs (20%).

In contrast to E. multilocularis, the larval tissue grows by displacing rather than infiltrating [3]. Consequently, it is easier to surgically remove or utilize minimally invasive procedures to address it, and the prognosis for CE is significantly better than that for alveolar echinococcosis (AE) [5,6]. The most commonly used minimally invasive technique is percutaneous aspiration, injection of chemicals, and reaspiration (PAIR). This procedure aims to destroy the germinal layer and is only suitable for CE1 and CE3a cysts; however, it carries risks such as anaphylaxis and recurrence [7,8]. Alternatively, to evacuate the entire endocyst, the modified catheterization technique (MocT) can be performed [5,8]. This method is reserved for large cysts or those with daughter vesicles (CE2, CE3b) and is associated with risks like secondary bacterial infection, abscess formation, or biliary fistulas. Both procedures are primarily performed in endemic areas, such as Turkey [9], while expertise is often lacking in non-endemic countries like Switzerland.

The therapeutic approach is stage-dependent, according to cyst type, size, location, and presence of complications. Management options include anthelmintic therapy (mainly albendazole), percutaneous or surgical intervention, or ultrasound-based monitoring.

Indications for surgery are traditionally linked to complications of cysts, including cyst rupture, biliary fistula, compression of vital structures, and secondary infection or hemorrhage [5,10]. Surgery is also required for managing cysts that possess multiple daughter vesicles and are unsuitable for percutaneous treatment (for example, WHO stage CE2 and CE3b) [5,10]. Additional reasons for surgery include a cyst diameter greater than 10 cm and superficial cysts that are at risk of rupture due to trauma [5]. In these cases, adjunctive drug therapy must be administered regularly to reduce the risk of secondary echinococcosis from seeding in the abdominal cavity in case of fluid spillage [11].

A prerequisite for curative treatment of cystic echinococcosis is the complete excision of the cyst, including all structural components—germinal, laminated, and adventitial layers. Incomplete removal, as seen in procedures such as endocystectomy, partial cystectomy, or various percutaneous approaches, poses a potential risk of recurrence. However, the optimal extent of surgical resection remains a subject of debate. It is still unclear whether formal hepatectomy is always necessary or if total cyst excision (pericystectomy) yields an equally effective outcome. Hepatectomy may reduce the risk of recurrence, yet it involves resecting a substantial volume of functionally intact liver parenchyma, thus increasing perioperative morbidity. In contrast, pericystectomy facilitates radical removal of the cyst, including the adventitial layer, while preserving a greater proportion of healthy liver tissue, representing a potential balance between efficacy and safety [12–14]. Recurrent echinococcosis after resection happens in 2–25 percent of cases [5,12,13,15], depending on the location and size of the cyst, as well as the surgeon’s experience.

This multicenter analysis aims to evaluate the safety and recurrence outcomes of surgical treatment for cystic echinococcosis.

## Methods

### Ethics statement

The study was conducted in accordance with the principles of the Declaration of Helsinki and approved by the Cantonal Ethics Committee Zurich (BASEC ID: 2024–01732). This project represents a nested analysis of the Zurich Echinococcosis Cohort Study (BASEC ID: 2020–00495). All cohort participants provided written informed consent during outpatient visits or by mail prior to inclusion.

### Study design

A retrospective cohort study was conducted on patients with cystic echinococcosis who underwent surgical resection at the University Hospital Zurich and its partner cantonal hospitals between 2004 and 2024. Thirty-seven patients were included.

The primary aim of this study was to analyze the morbidity, mortality, and recurrence risk associated with the surgical treatment of cystic echinococcosis. The outcome measures included the Clavien-Dindo classification of complications, the Comprehensive Complication Index (CCI) at discharge, length of hospital stay, 90-day mortality, overall survival, and recurrence rate [16–18]. The secondary objectives were to assess factors influencing recurrence and complications, such as patient comorbidities, surgical techniques, cyst size, cyst number, and biliary involvement. Inclusion criteria consisted of radiologically confirmed *E. granulosus* infection with WHO stage classification CE 1-3b. The indication for surgery was made within a multidisciplinary framework involving visceral surgeons, infectious disease specialists, radiologists, and hepatologists. Decisions regarding operative versus non-operative management were based on cyst stage, anatomical characteristics, symptom burden, response to antiparasitic therapy, and overall patient risk profile, in accordance with contemporary guideline recommendations (WHO-IWGE). Patients managed non-surgically within the same multidisciplinary framework were not included, as the present study was designed specifically to evaluate outcomes following surgical treatment.

**Fig 1:**
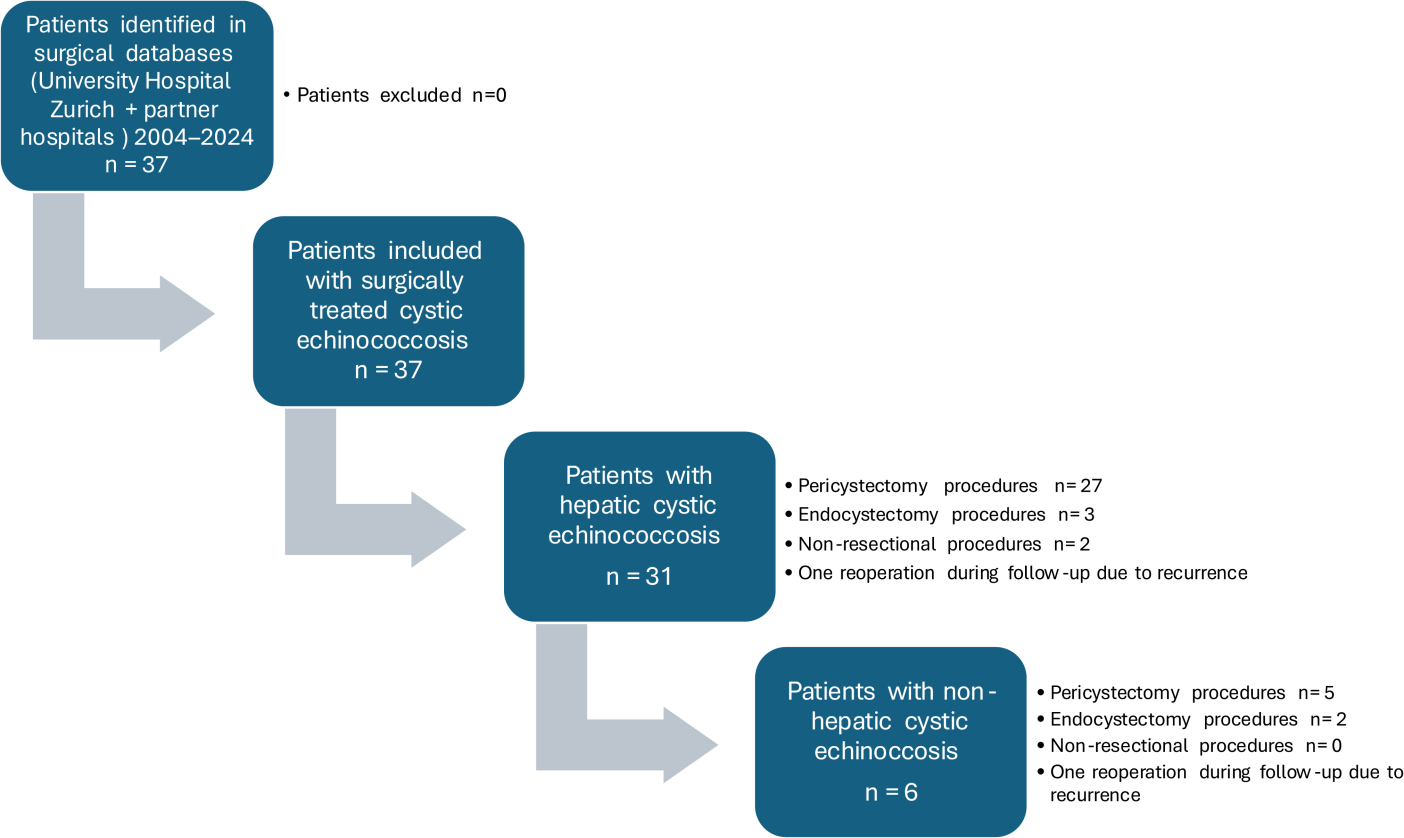
Study flow diagram of patient inclusion in the retrospective cohort of surgically treated cystic echinococcosis (2004-2024), including stratification into hepatic and non-hepatic disease and corresponding surgical techniques.

Analyses were performed at both the patient level (demographic characteristics, survival) and the procedure level (operative characteristics, perioperative outcomes, recurrence). Denominators are specified accordingly throughout the manuscript. Patient inclusion and procedural allocation are illustrated in Fig 1.

The diagnosis of cystic echinococcosis was established using serologic testing via enzyme-linked immunosorbent assay (ELISA) and imaging studies such as ultrasonography (US) and computed tomography (CT). Serology was supportive but not universally positive. CT imaging evaluated vascular involvement, while magnetic resonance imaging (MRI) was employed during the preoperative evaluation of liver disease to assess biliary tract involvement. Radiological assessments documented cyst number, size, and WHO classification. Data extracted from patient records included patient characteristics (sex, age, cardiac, pulmonary, and renal comorbidities, bleeding disorder, nicotine use, Eastern Cooperative Oncology Group (ECOG) performance status, and American Society of Anesthesiologists (ASA) classification), disease characteristics (cyst size, cyst number, biliary involvement, vascular compression on computed tomography imaging, anthelmintic medication, and its duration), as well as surgical data (technique, duration, blood loss, Pringle maneuver, water-jet dissection, irrigation with saline or glucose solution, cyst opening, CCI at discharge, and 90-day mortality). Preoperative ERCP was not performed in this patient cohort. Intra-operative drains were placed selectively at the discretion of the operating surgeon, particularly in cases with risk of bile leakage or large residual cavities.

Comorbidities were defined as diagnoses related to organ dysfunction, regardless of whether pharmacological treatment was given. Cardiac comorbidities included arterial hypertension, coronary artery disease, and dysrhythmias. Pulmonary comorbidities included chronic obstructive pulmonary disease, restrictive lung diseases, and previous lung resections that led to reduced respiratory capacity. Renal comorbidities were identified as chronic kidney disease with a reduced glomerular filtration rate. Recurrence was defined at the procedure level as the appearance of new active cysts after treatment, including regrowth of live cysts at the site of a previously treated cyst or the development of new distant disease due to cyst spillage.

Postoperative management and follow-up were standardized and conducted routinely. At our center, albendazole is generally administered starting at least one week prior to surgery and continued for a minimum of three months postoperatively, with a standard dosage of 15 mg/Kg/day. Mebendazole was given as an alternative in four patients due to asymptomatic elevation of the liver enzymes. The protocol includes serologic testing (ELISA) and abdominal ultrasonography at six months, one year, and annually thereafter in the absence of recurrence. A minimum follow-up of 10 years is performed at our center unless the patient declines. Any suspicion of recurrence identified on ultrasonography is confirmed with CT imaging and first managed with albendazole or mebendazole.

### Surgery

Pericystectomy and endocystectomy were the two resective techniques used to surgically address the hepatic CE cyst. The choice between pericystectomy and endocystectomy was determined during multidisciplinary evaluation based on cyst characteristics, including size, anatomical location, proximity to major vascular or biliary structures, presence of complications, and anticipated operative risk. Pericystectomy was preferred whenever complete cyst excision could be achieved safely while preserving functional liver parenchyma. Endocystectomy was selected in cases where radical resection was considered technically unsafe or associated with an increased risk of vascular or biliary injury.

Pericystectomy is usually performed with the assistance of a parenchyma dissection device such as CUSA or Water-Jet. In this technique, surgeons operate along the outer layer of the cyst and dissect the hepatic parenchyma away from it using these tools. This can achieve complete clearance of the cyst from pedicles without the need for primary liver resection, resulting in maximal parenchyma sparing. Pericystectomy is, by definition, associated with technically R0 resection margins if performed correctly, as the dissected parenchyma provides a safety margin of a few millimeters. On the other hand, endocystectomy involves surgically opening the cyst with precautionary measures placed around the liver (e.g., gauzes saturated with hypertonic saline) to prevent content contamination. After the cyst is opened, the contents are carefully aspirated, and the cavity is irrigated with saline. This may be followed by enlarging the opening and removing the germinal layer to prevent recurrence and eliminate any infectious content. This surgery is, by definition, not an R0 procedure because the outermost layer of the CE cyst still remains in situ and is therefore associated with a certain risk of recurrence.

Non-resectional procedures were only performed in cases where the cyst was in close proximity to major vascular or biliary structures, rendering resection unsafe.

### Statistical analysis

All data were recorded using IBM SPSS software, version 26 for Mac (IBM Corp., Armonk, NY, USA). A descriptive analysis of patient characteristics (sex, age, cardiac, pulmonary, and renal comorbidities, bleeding disorders, nicotine use, ECOG performance status, and ASA classification), disease characteristics (cyst size, cyst number, biliary involvement, vascular compression on computed tomography imaging, anthelmintic medication, and its duration), and surgical data (technique, duration, blood loss, Pringle maneuver, water-jet dissection, irrigation with saline or glucose solution, cyst opening, CCI at discharge, and 90-day mortality) was performed. A subgroup analysis was also conducted for patients with hepatic disease.

For the primary outcomes, postoperative complications were analyzed using the Clavien-Dindo classification and the Comprehensive Complication Index (CCI), reported as frequencies and medians, respectively. Recurrence rates were calculated as proportions and stratified by surgical technique. Overall survival was analyzed using the Kaplan-Meier survival curve. Exact binomial 95% confidence intervals were calculated for key outcome proportions using the Clopper–Pearson method**.**

All analyses were descriptive and exploratory in nature, reflecting the limited sample size and number of events.

## Results

### Patient demographics

The majority of patients, 62.2% (n = 23), were male, with a median age of 45 years (28–80). A total of 86.5% (n = 32) of patients came from echinococcosis-endemic areas (Kosovo, Macedonia, Tunisia, Turkey, Italy, Bulgaria, Montenegro, Syria, China), and eight (21.6%) had previously undergone parenchyma-sparing surgery for CE in their respective countries before arriving in Switzerland. Cardiac comorbidities were the most frequently observed (18.9%), followed by renal comorbidities (10.8%) and pulmonary comorbidities (8.1%). Seventeen patients (46.0%) were active smokers at the time of surgery. The ECOG performance status was < 2 in 92.1% of patients, and the ASA score was predominantly 2 (84.2%). The demographic characteristics of all patients, including a separate analysis for those with hepatic cystic echinococcosis, are presented in Table 1.

**Table 1:** Demographic characteristics of the patient population.

	All patients N = 37	Patients with hepatic cystic echinococcosis N = 31
**Sex**		
Male	23 (62.2%)	18 (58.1%)
Female	14 (37.8%)	13 (41.9%)
**Age (median)**	45 (28–80)	45 (28-80)
**Country of Origin**		
Kosovo	10 (27.0%)	7 (22.6%)
Macedonia	6 (16.2%)	6 (19.4%)
Tunisia	2 (5.4%)	2 (6.5%)
Turkey	7 (18.9%)	7 (22.6%)
Italy	1 (2.7%)	1 (3.2%)
Bulgaria	1 (2.7%)	1 (3.2%)
Montenegro	1 (2.7%)	1 (3.2%)
Switzerland	5 (13.5%)	3 (9.7%)
Syria	3 (8.1%)	3 (9.7%)
China	1 (2.7%)	0 (0.0%)
**Cardiac Comorbidities**	7 (18.9%)	6 (19.4%)
**Pulmonary Co-morbidities**	3 (8.1%)	1 (3.2%)
**Renal Comorbidities**	4 (10.8%)	3 (9.7%)
**Bleeding disorder**	2 (5.4%)	2 (6.5%)
**Smoking status**		
Current smokers	17 (46.0%)	15 (48.4%)
Past smokers	1 (2.7%)	1 (3.2%)
Non-smokers	19 (51.4%)	15 (48.4%)
**ECOG**		
0	26 (70.2%)	25 (80.7%)
1	8 (21.6%)	5 (16.1%)
2	2 (5.4%)	1 (3.2%)
3	1 (2.7%)	0 (0.0%)
4	0 (0.0%)	0 (0.0%)
5	0 (0.0%)	0 (0.0%)
**ASA**		
1	3 (7.9%)	3 (9.7%)
2	32 (84.2%)	27 (87.1%)
3	3 (7.9%)	2 (6.5%)

### Disease characteristics

Of the 37 patients included, 83.8% (n = 31) had hepatic involvement, 8.1% (n = 3) had pulmonary involvement, and 8.1% (n = 3) had disease in other locations (two cases in the intestine and one case in the psoas compartment). Most patients had one (69.2%, n = 27) or two (12.8%, n = 5) cysts, and the median cyst size was 8 cm (2.1–19 cm), both overall and in cases of liver disease. All patients received antiparasitic treatment perioperatively, either with albendazole (89.7%) or mebendazole (10.3%). The median duration of perioperative anthelmintic treatment was 4 months (2–204). In 82.1% of the 39 surgical interventions (n = 32), the number of cysts was 1 or 2, and the median cyst size was 8 cm (2.1–19 cm). Biliary tract compression was observed in 6.3% (n = 2) of hepatic disease cases, and vascular compression (of the right hepatic vein, middle hepatic vein, and/or inferior vena cava) was noted in 18.8% (n = 6) of liver surgery cases. The data regarding disease characteristics are presented in Table 2.

**Table 2:** Disease characteristics of the patient population.

	All procedures N = 39	Liver surgery N = 32
**Organ affected**		
Liver	32 (82.1%)	32 (100.0%)
Lung	4 (10.3%)	0 (0.0%)
Other locations	3 (7.7%)	0 (0.0%)
**Cyst number**		
1	27 (69.2%)	21 (65.6%)
2	5 (12.8%)	5 (15.6%)
3	3 (7.7%)	3 (9.4%)
4	1 (2.6%)	0 (0.0%)
>5	3 (7.7%)	3 (9.4%)
**Cyst size (median, cm)**	8 (2.1 - 19)	8 (3– 19)
**Biliary tract compression**	2 (5.1%)	2 (6.3%)
**Vascular compression**	6 (15.4%)	6 (18.8%)
**Preoperative anthelmintic treatment**		
with albendazole	35 (89.7%)	28 (87.5%)
with mebendazole	4 (10.3%)	4 (12.5%)
**Duration of the anthelmintic treatment (median, months)**	4 (2 – 204)	3 (2 – 94)

### Surgery

Open pericystectomy was the most commonly performed procedure overall, accounting for 82.1% (n = 32), followed by open endocystectomy at 12.8% (n = 5). In the hepatic disease group (n = 32), 27 pericystectomies were carried out. Among these, 16 procedures were limited resections, 2 were bisegmentectomies, 6 were right hepatectomies, and 3 were left hepatectomies. For all procedures, an R0 resection rate of 93.8% was observed, with a rate of 96.3% in the liver surgery group (see Table 3). In the two R2 cases, no disease remained in the operated organ (liver or lung); however, the disease was present in another anatomical region, deemed inoperable, specifically in the cervical spine in one case and the pelvic area in the other. In the first case, a 9.4 cm cyst in the liver necessitated a bisegmentectomy of segments IVa and VIII due to its size and location. In the second case, a 12 cm cyst at the superior thoracic aperture was surgically addressed because of its compressive effects and anatomical complexity. The median duration of surgery was 307.5 minutes (100–600 minutes), and the median blood loss was 290 ml (50–2000 ml). The Pringle maneuver was performed in 43.8% (n = 14) of liver cases, with a documented total duration ranging between 15 and 60 minutes. In the liver group, dissection was conducted using a water-jet technique in 46.9% of cases (n = 15). A controlled cyst opening rate of 43.6% was observed overall, with the liver surgery group displaying a similar rate of 40.6%. Surgeons utilized a hyperosmolar saline solution of 40% in 30.8% (n = 12) of all cases or a 40% glucose solution in 7.7% (n = 3) of all cases. All surgical parameters are presented in Table 4.

**Table 3:** Resection Status.

	All resective procedures (n = 32)	Hepatic resective procedures (n = 27)
**Resection Status**		
R0	30 (93.8%)	26 (96.3%)
R2	2 (6.3%)	1 (3.7%)

**Table 4:** Surgical parameters.

	All procedures N = 39	Liver surgery N = 32
**Type of surgery/intervention**		
Endocystectomy	5 (12.8%)	3 (9.4%)
Cyst aspiration and fenestration	1 (2.6%)	1 (3.1%)
Open PAIR and cyst deroofing	1 (2.6%)	1 (3.1%)
Pericystectomy	32 (82.1%)	27 (84.4%)
**Intraoperative cyst opening**	17 (43.6%)	13 (40.6%)
**Irrigation with hyperosmolar saline solution**	12 (30.8%)	11 (34.4%)
**Irrigation with 40% glucose solution**	3 (7.7%)	2 (6.3%)
**Extent of Liver Resection**		
Limited Resection (1 segment or less)	11 (28.2%)	16 (50%)
Bisegmentectomy	2 (5.13%)	2 (6.3%)
Trisegmentectomy	0 (0.0%)	0 (0.0%)
Left Hemihepatectomy	3 (7.7%)	3 (9.4%)
Right Hemihepatectomy	6 (15.4%)	6 (18.8%)
**Duration of the procedure (median, minutes)**	307.5 (100 – 600)	307.5 (142 – 600)
**Blood loss (median, ml)**	290 (50-2000)	290 (95-2000)
**Pringle maneuver**	14 (35.9%)	14 (43.8%)
**Water-jet dissection**	15 (38.5%)	15 (46.9%)

### Postoperative course

Fifteen patients (38.5%) were transferred to the intensive care unit postoperatively. The median length of hospital stay was 8 days (5–23). Overall complications occurred in 43.2% (n = 16, 95% confidence interval 27.1–60.5) of cases; however, only 8.3% (n = 2) of all complications (n = 24) were severe (Clavien-Dindo IIIb). The median Comprehensive Complication Index (CCI) was 8.7 (0 – 47.2) at discharge. Reported complications included pneumothorax requiring chest tube insertion, pneumonia, urinary tract infection, bleeding necessitating transfusion, perihepatic seroma requiring percutaneous drainage, exudative pancreatitis, pulmonary edema, atrial flutter, wound infection, and paralytic ileus. Iatrogenic pneumothorax and pneumonia were the most common complications. No relaparotomy was necessary in the acute postoperative period. Accidental intraoperative cyst perforation occurred in one case, as did intraoperative diaphragmatic perforation in another case. See Table 5 for a detailed description of the reported postoperative complications. The 90-day mortality rate was 0%, and the only fatality in the cohort occurred four years later due to complications from a heart transplant. Table 6 provides further information regarding primary and secondary outcomes.

**Table 5:** Reported postoperative complications.

Postoperative Complication	Number of events reported
Iatrogenic pneumothorax	3
Pneumonia	3
Pulmonary embolism	1
Urinary tract infection	1
Bleeding	1
Perihepatic seroma	1
Exudative pancreatitis	1
Pulmonary edema	1
Atrial flutter	1
Wound infection	1
Paralytic ileus	2
Accidental intraoperative cyst perforation	1
Intraoperative diaphragmatic perforation	1

**Table 6:** Primary and secondary outcomes.

	All procedures N = 39	Liver surgery N = 32
**Stay at the intensive care unit**	15 (38.5%)	12 (37.5%)
**Length of stay (median, days)**	8 (5 -23)	8 (5 – 23)
**Clavien-Dindo Classification**		
Grade 1	6	4
Grade 2	13	10
Grade 3a	3	3
Grade 3b	2	1
Grade 4	0	0
**CCI at discharge (median)**	8.7 (IQR 0-25.3)	8.7 (IQR 0-24.6)
**90-day mortality rate**	0/39 (0%; 95% CI 0–9.0)	0/32 (0%; 95% CI 0.0–10.9)
**Median Overall survival (months)**	93 (12-230)	78 (12-230)
**Overall Recurrence**	3/39 (7.7%; 95% CI 1.6–20.9)	2/32 (6.3%; 95% CI 0.8–20.8)
**Recurrence per operative procedure**		
**-** Endocystectomy	1	0
- Cyst aspiration and fenestration	1	1
- Open PAIR and cyst Deroofing	1	1
- Pericystectomy	0	0

### Follow-up

The median follow-up time was 93 months (12–230 months). During this period, three cases of recurrence (7.7%) were identified, necessitating repeated resection in two of the cases. One patient, who had originally undergone an endocystectomy via thoracotomy, required a second thoracotomy with pericystectomy five years postoperatively. Another patient, who had gone through open cyst aspiration and fenestration, developed a new retention cyst in liver segments VII/VIII, which compressed the vena cava. Consequently, the patient needed a relaparotomy with right hemihepatectomy seven months later. In the final case, a hepatic recurrence was noted three months after open PAIR and cyst deroofing, which was treated pharmacologically with albendazole.

**Fig 2:**
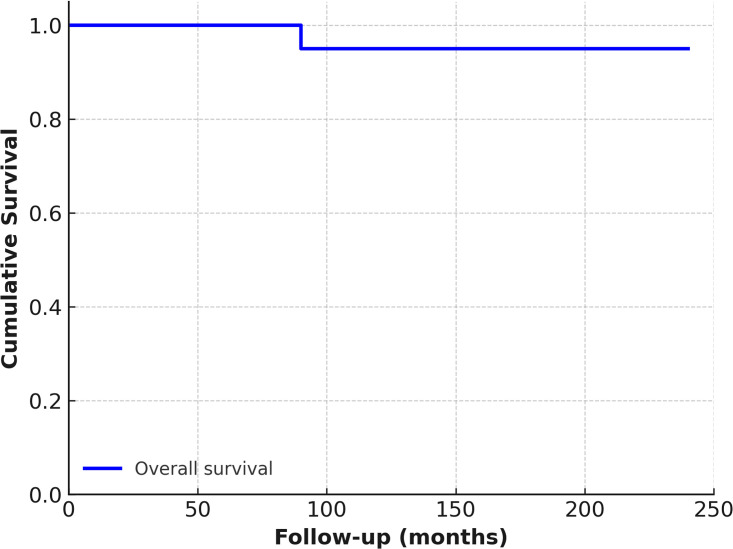
Kaplan–Meier estimate of overall survival in patients undergoing surgical treatment for cystic echinococcosis over the follow-up period.

**Fig 3:**
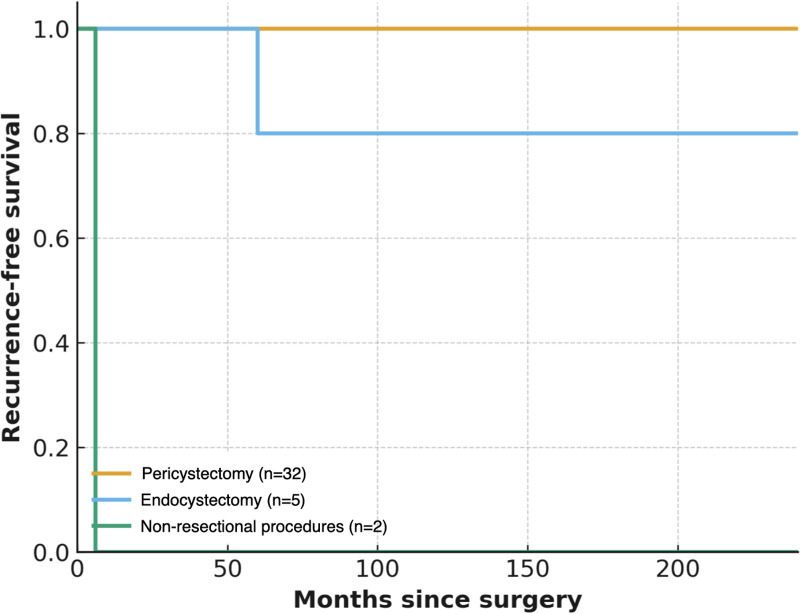
Kaplan–Meier estimate of recurrence-free survival in patients undergoing surgical treatment for cystic echinococcosis stratified by surgical technique over the follow-up period.

Overall survival was calculated using the Kaplan–Meier analysis (Fig 2), while recurrence-free survival was stratified by surgical technique (Fig 3).

## Discussion

This retrospective cohort study examines 20 years of surgical management for cystic echinococcosis at a Swiss tertiary referral center and partner cantonal hospitals, offering significant insights into long-term outcomes in a non-endemic environment. Among the 37 patients who underwent surgical treatment, 83.8% had liver involvement, with most receiving pericystectomy or endocystectomy. The overall recurrence rate was 7.7% (n = 3), occurring after three different procedures (endocystectomy, cyst aspiration and fenestration, and open PAIR with cyst deroofing). No recurrences were observed in the pericystectomy group. Postoperative complication rates were low, with no 90-day mortality reported. These findings suggest favorable long-term outcomes following surgical treatment in a non-endemic setting and support its continued role in carefully selected patients [19–21]. However, given the limited sample size and low number of recurrence events, the present analysis should be interpreted as descriptive and exploratory rather than comparative.

In contemporary practice, management of cystic echinococcosis requires an interdisciplinary approach integrating surgical, infectious disease, radiological, and hepatological expertise. Surgery represents only one component within a broader treatment spectrum that includes antiparasitic therapy and imaging-based surveillance. The present study therefore reflects surgical outcomes within a multidisciplinary care model rather than advocating surgery as a standalone treatment strategy.

For CE, the primary surgical techniques are pericystectomy and endocystectomy, each presenting unique benefits and drawbacks. Pericystectomy involves the complete excision of the hydatid cyst, including both the parasitic components and the host-derived adventitial layer. It is classified as a radical procedure and has been consistently associated with lower recurrence rates and better long-term outcomes. Cirenei et al. noted a recurrence rate of just 0.9% following radical procedures like pericystectomy or liver resection, as opposed to 11.2% for patients receiving conservative surgery such as endocystectomy, which is consistent with findings from a more recent systematic review and meta-analysis [22,23]. Rinaldi et al. further emphasized that pericystectomy significantly reduces the risk of postoperative complications related to residual cavity infections or biliary fistulas, due to the thorough removal of infected and inflamed tissues [24]. Govindasamy et al. also observed that recurrence is minimized when the entire pericyst is excised, particularly by preventing exogenous vesiculation and secondary cyst formation [25]. However, pericystectomy remains technically demanding and carries increased intraoperative risk, especially when cysts are attached to major vascular or biliary structures [24].

Endocystectomy is a parenchyma-sparing technique that removes the internal parasitic germinal layer while retaining the fibrous host capsule, which may contain viable scolices and contribute to recurrence. A recent systematic review encompassing 54 studies (n = 4.058 patients) indicated a recurrence rate of 4.8% and a mortality rate of 1.2% for endocystectomy, with a higher likelihood of recurrence if cavity management is not optimal [26]. Although these outcomes may be considered acceptable, particularly in resource-limited settings or anatomically complex situations, recurrence rates reported for endocystectomy are higher than those described for pericystectomy in the literature. The elevated recurrence risk of endocystectomy has been associated with exogenous vesiculation and the challenges of ensuring complete removal of germinal debris from the residual cavity [26].

A valuable adjunct to pericystectomy that enhances safety and precision is the use of water-jet dissection. This technique facilitates selective parenchymal dissection while protecting biliary and vascular structures, which significantly reduces the risk of bleeding and bile leaks. In our institution’s experience, water-jet dissection enabled *en bloc* removal of the cyst with minimal tissue damage, especially in lesions that are centrally or perivascularly located. Although prospective comparative studies are limited, emerging evidence highlights the utility of water-jet dissection in hepatobiliary surgery for CE, particularly in enhancing the thoroughness of pericystectomy while maintaining surgical safety.

Future developments in CE management increasingly focus on minimally invasive strategies, including laparoscopic and robot-assisted surgery, as well as image-guided percutaneous and thermal ablation techniques. While these approaches are promising and may reduce postoperative morbidity in selected cases, they require strict patient selection and substantial expertise. The risk of cyst rupture and spillage remains a major concern, particularly in minimally invasive settings, and these techniques should therefore be restricted to specialized referral centers with experience in CE surgery [27,28].

Importantly, surgery is not indicated for all patients with CE. Operative treatment should generally be avoided in patients with inactive or calcified cysts (CE4–CE5), asymptomatic uncomplicated cysts suitable for medical therapy or watch-and-wait strategies, patients with diffuse multiorgan disease, or those with prohibitive operative risk. These considerations underscore the necessity of stage-adapted, multidisciplinary decision-making based on cyst morphology, location, patient comorbidities, and available expertise [28].

Surgical treatment of CE requires specific training and experience beyond that needed for simple cystic liver lesions. Inadequate technique may lead to intraoperative spillage of viable protoscolices, secondary echinococcosis, and severe complications, including anaphylaxis. Safe surgical management demands familiarity with parasite biology, meticulous handling of the cyst, use of scolicidal agents, and appropriate strategies to prevent dissemination. Concentration of surgical CE management in specialized referral centers is therefore essential to optimize outcomes and minimize preventable complications [28].

An important strength of this study is the long duration of follow-up, with a median observation time of 93 months. Recurrence of cystic echinococcosis may occur many years after treatment, and studies with limited follow-up may therefore underestimate the true recurrence rate. The extended follow-up available in this cohort provides valuable insight into the long-term durability of surgical management and supports the role of surgery as part of multidisciplinary care in non-endemic healthcare settings.

In conclusion, optimal CE treatment should be multimodal and tailored to individual patient characteristics. Radical pericystectomy may represent an appropriate surgical strategy for suitable patients, owing to its superior long-term outcomes and significantly lower recurrence rates in the literature. Endocystectomy may still be necessary for high-risk patients or when radical resection is unfeasible due to anatomical constraints. Adjunctive techniques like water-jet dissection enhance the safety and feasibility of pericystectomy and should be utilized in complex cases. Antiparasitic therapy with albendazole remains essential before and after surgery. Ultimately, a multidisciplinary and staged approach - including surgery, medical therapy, and, where appropriate, endoscopic or percutaneous methods - provides the best opportunity for durable disease control and minimizing recurrence in CE.

## Conclusion

Surgical therapy continues to play an important role in the management of selected cases of cystic echinococcosis. In this long-term cohort, surgical treatment was associated with low perioperative morbidity and no procedure-related mortality. Surgery enables complete removal of the parasitic cyst, including the germinal layer, within a single intervention and has been associated with low recurrence rates in the literature. In this cohort, no recurrences were observed after pericystectomy; however, the limited number of events precludes formal comparison between surgical techniques.

### Limitations

A key limitation of this retrospective study is potential selection bias, as non-surgically treated patients were not included. Variability in surgical techniques and postoperative management across the study period further limits comparability and conclusions regarding the most effective approach. In addition, the relatively small cohort size and low number of outcome events limit statistical precision and preclude formal comparison between surgical techniques. Follow-up duration was inherently influenced by the timing of surgery, with more recently treated patients still undergoing active surveillance and therefore contributing shorter observation periods than earlier cases, which may lead to differential opportunities for recurrence detection and introduce potential survivor bias. Accordingly, the findings should be interpreted within the context of a descriptive observational cohort.

## Supporting information

S1 Data De-identified dataset underlying the analyses presented in this study. The dataset includes patient demographics, disease characteristics, surgical parameters, postoperative outcomes, and follow-up data for patients undergoing surgical treatment of cystic echinococcosis.
(XLSX)
